# Risk factors for peripheral intravenous catheter-related phlebitis in adult patients^*^

**DOI:** 10.1590/1980-220X-REEUSP-2021-0398en

**Published:** 2022-06-10

**Authors:** Adriana Moreira Noronha Simões, Patrícia Vendramim, Mavilde Luz Gonçalves Pedreira

**Affiliations:** 1 Universidade Federal de São Paulo, São Paulo, SP, Brazil.; 2 Universidade Federal de São Paulo, Escola Paulista de Enfermagem, São Paulo, SP, Brazil.

**Keywords:** Phlebitis, Peripheral Catheterization, Adult, Nursing, Flebitis, Cateterismo periférico, Adulto, Enfermería, Flebite, Cateterismo periférico, Adulto, Enfermagem

## Abstract

**Objective:**

To identify risk factors for peripheral intravenous catheter-related phlebitis in adult patients.

**Method:**

This is a *post hoc* analysis of a randomized clinical trial, totaling 1,319 patients. Demographic and clinical variables related to therapy and phlebitis were investigated. For data analysis, frequencies, measures of central tendency and dispersion were calculated, and Pearson’s chi-square test and Fisher’s exact test were used, with logistic regression, ROC curve, and *Odds Ratio* calculation (95% confidence interval; 5% significance level) being implemented.

**Results:**

Of the 1,319 participants, 80 (6.1%) developed phlebitis. The following were associated with the occurrence of phlebitis: reduced mobility (p = 0.015), family history of deep vein thrombosis (p = 0.05), catheterization of veins on the back of the hand (p = 0.012), pain (p < 0.01), Amoxicillin-Potassium Clavulanate (p = 0.015), and Omeprazole Sodium (p = 0.029).

**Conclusion:**

Risk factors for phlebitis involved intrinsic and extrinsic factors to the patient, indicating preventive nursing interventions such as promoting patient mobility, not catheterizing veins in the dorsal arch of the hand, cautious infusion of risk drugs, and valuing pain complaints.

## RESUMO

**Objetivo:**

Identificar fatores de risco para flebite relacionada a cateteres intravenosos periféricos em pacientes adultos.

**Método:**

Estudo de análise *post hoc* de ensaio clínico randômico, totalizando 1.319 pacientes. Pesquisaram-se variáveis demográficas, clínicas, relacionadas à terapia e à flebite. Para análise de dados, calcularam-se frequências, medidas de tendência central e dispersão, e utilizaram-se os testes Qui-quadrado de Pearson e Exato de Fisher, implementando-se regressão logística, curva ROC e cálculo de *Odds Ratio* (intervalo de confiança 95%; nível de significância 5%).

**Resultados:**

Dos 1.319 participantes, 80 (6,1%) desenvolveram flebite. Associaram-se à ocorrência de flebite mobilidade reduzida (p = 0,015), história familiar de trombose venosa profunda (p = 0,05), cateterização de veias do dorso da mão (p = 0,012), dor (p < 0,01), Amoxicilina-Clavulanato de Potássio (p = 0,015) e Omeprazol sódico (p = 0,029).

**Conclusão:**

Os fatores de risco para flebite envolveram fatores intrínsecos e extrínsecos ao paciente, indicando intervenções de enfermagem preventivas, como promover a mobilidade do paciente, não cateterizar veias do arco dorsal da mão, infusão cautelosa de fármacos de risco e valorizar a queixa de dor.

## RESUMEN

**Objetivo:**

Identificar factores de riesgo de flebitis relacionada a catéteres intravenosos periféricos en pacientes adultos.

**Método:**

Estudio de análisis *post hoc* de ensayo clínico randómico, totalizando 1.319 pacientes. Se investigaron variables demográficas, clínicas, relacionadas a la terapia y a la flebitis. Para análisis de datos se calcularon frecuencias, medidas de tendencia central y dispersión y las pruebas Chi-cuadrado de Pearson y Exacto de Fisher, se implementó con regresión logística, curva ROC y cálculo de *Odds Ratio* (intervalo de confianza 95%; nivel de significancia 5%).

**Resultados:**

De los 1.319 participantes, 80 (6,1%) desarrollaron flebitis. Se asociaron a la ocurrencia de flebitis: movilidad reducida (p = 0,015), historial familiar de trombosis venoso profundo (p = 0,05), cateterización de venas del dorso de la mano (p = 0,012), dolor (p < 0,01), Amoxicilina-Clavulanato de Potasio (p = 0,015) y Omeprazol sódico (p = 0,029).

**Conclusión:**

Los factores de riesgo para flebitis involucraron factores intrínsecos y extrínsecos al paciente, indicando intervenciones de enfermería preventivas como promover la movilidad del paciente, no cateterizar venas del arco dorsal de la mano, infusión cautelosa de fármacos de riesgo y valoración de la queja de dolor.

## INTRODUCTION

It is estimated that more than 80% of hospitalized patients require intravenous therapy (IVT)^(1)^, implemented mainly with the use of peripheral intravenous catheters (PIC), since they provide quick access to the vascular system, being the most performed invasive intervention in the health area, and less invasive and more cost-effective than other types of intravenous catheters^(2–4)^. Thus, due to the diagnostic and therapeutic scope of IVT, the insertion of PIC is one of the most frequent interventions in clinical nursing practice^(1,5,6)^.

Phlebitis is one of the PIC-related complications in adults, causing discomfort to the patient, interruption of therapy, higher care costs, being able to result in negative patient’s experience with the nursing practice^(7,8)^. It is an inflammatory process of the vein intimal layer, of varying severity, classified in mechanical phlebitis when it results, especially, from the trauma to the vessel wall caused by PIC; in chemical phlebitis, when resulting from the infusion of fluids or solutions with extremes of pH and osmolarity, irritating to vascular tissue or not adequately solubilized; and in infectious phlebitis when related to breach of aseptic technique during PIC insertion or maintenance from handling dressings, devices, and solutions^(9)^. It should also be noted that there is the possibility of identifying post-infusion phlebitis, when the complication is identified up to 48 hours after the removal of PIC^(10)^.

Clinical manifestations include edema, hyperemia, pain, local heat, fibrous cord along the vessel path and, in cases of infection, drainage of purulent exudate may occur at the PIC insertion site^(11)^. It can also be categorized according to severity, with the use of scales being suggested. The Infusion Nursing Society (INS) Phlebitis Scale is the most commonly applied in Brazil, ranging from 0 to 4, with zero being the absence of complications, progressing according to the symptoms and signs of inflammation, pointing to greater severity of phlebitis, up to grade 4 which covers indicators of the presence of infection^(1)^.

The results of studies^(8,12–14)^ investigating risk factors related to the occurrence of this complication are diverse and divergent, pointing to multifactorial demographic, clinical, and IVT-related causes. In this study, we sought to investigate the occurrence of PIC-related phlebitis, to expand knowledge about the occurrence of this complication and to create subsidies capable of promoting decision-making and nursing interventions favoring its prevention, supporting care practice, and aiming to reduce IVT-related damage, since the identification of risk factors can help in the development of protocols and the institution of care directed to the patient’s individuality, to prevent the occurrence of the phlebitis complication. Thus, this study aimed to assess risk factors for the development of PIC-related phlebitis in adult patients.

## METHOD

### Design of Study

This is a study characterized as *post hoc* analysis of a randomized, controlled, non-inferiority clinical trial (RCT) on the influence of the PIC withdrawal method on the occurrence of phlebitis, called “Removal of peripheral intravenous catheters according to clinical signs of every 96 hours: a randomized, controlled, and non-inferiority study” and represented by the acronym *ResPeCt,* based on its title^(15)^.

The ResPeCt Study, a randomized, controlled, non- inferiority clinical study, aimed to check if PIC removal on clinical indication was not inferior to scheduled removal every 96 hours regarding the occurrence of phlebitis and to compare the severity of phlebitis, PIC length of stay, and other complications of intravenous therapy among study groups. In the primary study, the dependent variable for the sample calculation was phlebitis, its prevalence being stipulated at 5%, in line with the recommendation of the INS (Infusion Nurses Society) and with a non-inferiority margin of 3%. A significance level of 5% and test power of 80% were also adopted. A sample of 1,305 patients was estimated. The final study sample consisted of 1,319 patients, with 672 (50.9%) patients with PIC removal due to clinical indication and 647 (49.1%) with PIC removed every 96 hours. The incidence rate of phlebitis/1000 PIC-day was 14.9 in the clinical indication group and 23.8 in the 96-hour removal group (p = 0.006). The study showed that withdrawal according to clinical indication was not inferior to withdrawal every 96 hours regarding the occurrence of phlebitis^(15)^. The primary study was included in a Cochrane systematic review on the occurrence of phlebitis associated with peripheral intravenous catheters, being considered robust to support such a review^(14)^.

### Sample

For the present investigation, the ResPeCt study participants who developed phlebitis were compared to those who did not, aiming at investigating the existence of a causal association with exposure to risk factors for the occurrence of this PIC-related complication.

The selected inclusion criteria were: age 18 years or older; patients with PIC in place in clinical, surgical, intensive care units, or operating room; estimated treatment plan of IVT performed by PIC for at least 96 hours, as well as acceptance of the proposals expressed in the Free Informed Consent Form by the patient or guardian. As exclusion criteria, the following were listed: medical diagnosis of Catheter-related Blood Stream Infection and/or sepsis; neutrophil count of less than or equal to 1000/mm^3^ and simultaneous use of more than one PIC.

The sample of the present investigation consisted of 1,319 patients. The cases were represented by 80 participants who developed phlebitis and the controls were the 1,239 patients with PIC who did not present the complication. The present investigation was carried out from March 2019 to October 2020.

### Study Variables

The dependent variable, the occurrence of PIC-related phlebitis, was measured by the categories, yes and no, as well as its severity classification, through the application of the Phlebitis Scale recommended by the INS in 2011 and the translation into Portuguese validated in 2016^(1,16)^. This Phlebitis scale is graded from zero to four, with each level corresponding to the signs and/or symptoms of phlebitis. Grade 0 means absence of phlebitis. Grade 1 considers the presence of erythema with or without local pain. Grade 2 defines the presence of erythema, with or without pain or swelling, with induration. Grade 3 means the presence of erythema, with or without pain or swelling with induration and a palpable fibrous cord. Grade 4 is scored when there is pain, with erythema and/or edema, with induration and a palpable venous cord greater than 2.5 cm in length, and purulent drainage^(1.16)^.

Demographic and clinical variables were also studied to categorize the participants, as well as identify their possible relationship with the occurrence of the dependent variable, namely: age, sex, skin color, body mass index (BMI), place of hospitalization (H1 and H2), type of admission, chronic diseases, risk for venous thromboembolism, family history of venous thromboembolism, presence of venous thromboembolism, use of oral antiplatelet agents, use of oral anticoagulants, use of subcutaneous (SC) anticoagulants, and reduced mobility.

PIC- and therapy-related variables were also studied for possible characterization as a risk factor for phlebitis, including PIC type and caliber, laterality of the punctured limb, insertion site, PIC method of use, administration of antibiotics (ATB) and other drugs. Such variables were studied as they were aspects of sample description and of possible clinical and therapeutic relationship to phlebitis^(12,13)^.

### Data Collection

This study began on March 2019, through a literature review on the factors associated with the occurrence of PIC-related phlebitis. Subsequently, the primary study database was examined and the variables of interest were selected, their data compiled into a Microsoft Excel^®^ spreadsheet, initiating the secondary analysis. It should be noted that the researchers also participated in the primary study and thus had access to data.

### Data Analysis and Treatment

For statistical analysis, the software R3.1.6 – 2009–2019 (RStudio, Inc.^©^) was used. Categorical variables were presented according to absolute and relative frequency; numerical variables according to mean, median, and measures of dispersion. For correlation analysis of categorical variables, Pearson’s Chi-Square and Fischer’s Exact tests were used.

For univariate and multivariable logistic regression analysis, the Chi-square Association Test was applied with the variables that fitted the model with p < 0.10 ep < 0.20, respectively.

The variables measure of effect was the *Odds Ratio* (OR) with a confidence interval (CI) of 95% and a significance level of 5%. The analysis of the PIC-related phlebitis occurrence model in the study sample was implemented through the Receiver Operating Characteristic Curve (ROC).

### Ethical Aspects

The RCT was registered on the platform *Clinical Trials* with the ID number NCT02568670, submitted to the Research Ethics Committee, and was approved. It also underwent a secondary analysis, which followed the standards of ethics in research involving human beings, according to Resolution No. 466/2012 of the National Health Council, and was approved by the Research Ethics Committee of the Universidade Federal de São Paulo, in 2020, with opinion number 4.161.802.

According to the inclusion criteria, and after clarification about the study, the RCT participants consented to participate in the research, formalizing the acceptance by signing the Free and Informed Consent Form.

## RESULTS

Of the 1,319 participants, 80 (6.1%) developed phlebitis and 1,239 (93.9%) did not present the complication. The most observed phlebitis grade was Grade II (48.7%), followed by grade I (40.0%), and grade III (11.3%). No cases of grade IV phlebitis nor catheter-related blood stream infection were identified.

As shown in Table 1, among the sample demographic and clinical characteristics, a difference was identified between the groups regarding the variables: place of hospitalization (p = 0.019), chronic diseases (p = 0.018), and reduced mobility (p = 0.011). A large part of the study sample was obtained from the elderly, white, overweight or obese people, with chronic diseases, at risk of VTE, reduced mobility, and admitted for surgical indication at the H1 health institution.

**Table 1.** Demographic and clinical characteristics of patients who did or did not develop peripheral intravenous catheter-related phlebitis – São Paulo, Brazil, 2020.

Demographic and Clinical Characteristics	Phlebitis				Total		p
	Yes		No				
	N	%	N	%	N	%
**Age (years)**							
Mean (±standard deviation)	59.79 (±20.48)						
**Age range**							0.919^a^
<30 years	6	7.5	109	8.7	115	8.7	
≥30 years to <60 years	30	37.5	489	39.5	519	39.3	
≥60 years to <80 years	28	35.0	391	31.6	419	31.8	
≥80 years	16	20.0	250	20.2	266	20.2	
**Sex**							0.338^a^
Female	36	45.0	626	50.5	662	50.2	
Male	44	55.0	613	49.5	657	49.8	
**Skin color** (n = 1,312)							0.705^b^
White	78	97.5	1,151	93.4	1229	93.7	
Brown	1	1.25	32	2.6	33	2.5	
Black	0	0.0	26	2.1	26	2.0	
Yellow	1	1.25	23	1.9	24	1.8	
**BMI** ^1^ (n = 1,295)							0.995^a^
Mean (±standard deviation)	26.48 (±5.43)						
Under weight	4	5.0	57	4.7	61	4.7	
Eutrophy	30	37.5	469	38.6	499	38.5	
Overweight	26	32.5	398	32.7	424	32.7	
Obesity	20	25.0	291	24.0	311	24.1	
**Place of hospitalization**							0.019^b^
H1	77	96.2	1,089	87.9	1166	88.4	
H2	3	3.8	150	12.1	153	11.6	
**Admission type** (n = 1,316)							0.710^a^
Clinical	11	13.7	189	15.3	200	15.2	
Surgical	69	86.3	1,047	84.7	1116	84.8	
**Chronic diseases**							0.018^a^
No	37	46.3	413	33.3	450	34.1	
Yes	43	53.7	826	66.7	869	65.9	
**VTE risk** ^2^							0.071^a^
No	14	17.5	330	26.6	344	26.1	
Yes	66	82.5	909	73.4	975	73.9	
**Family history of VTE** ^2^							0.171^b^
No	79	98.7	1,237	99.8	1316	99.8	
Yes	1	1.3	2	0.2	3	0.2	
**VTE** ^2^							1.000^b^
No	75	93.8	1,162	93.8	1237	93.8	
Yes	5	6.2	77	6.2	82	6.2	
**Use of oral antiaggregant**							0.559^a^
No	69	86.3	1,038	83.8	1107	83.9	
Yes	11	13.8	201	16.2	212	16.1	
**Use of oral anticoagulants**							0.814^b^
No	76	95.0	1.158	93.5	1234	93.6	
Yes	4	5.0	81	6.5	85	6.4	
**Use of SC anticoagulant** ^3^							0.292^b^
No	49	61.3	684	55.2	733	55.6	
Yes	31	38.8	555	44.8	586	44.4	
**Reduced mobility**							0.011^a^
No	48	60.0	906	73.1	954	72.3	
Yes	32	40.0	333	26.9	365	27.7	

^a^ Chi-square independence test, ^b^Fisher’s exact test, ^1^Body mass index, ^2^Venous thromboembolism, ^3^Subcutaneous.

Regarding the variables related to PIC and IVT (Table 2) there was a difference between the groups regarding pain complaints in patients who developed phlebitis. The predominant use of the 22 Gauge Nexiva^®^ catheter was identified, installed in the right forearm for intermittent infusion of antibiotics. Due to the primary study design, there was a similar proportion of PIC removed every 96 hours or as clinically indicated.

**Table 2.** Characteristics of peripheral intravenous catheter (PIC) and intravenous therapy (IVT) of patients who developed or did not develop peripheral intravenous catheters-related phlebitis – São Paulo, Brazil, 2020.

PIC and IVT Characteristics	Phlebitis				Total		p
	Yes		No				
	N	%	N	%	N	%
**Catheter type** (n = 1,228)							0.201^a^
Nexiva^®^	53	73.6	722	62.5	775	58.8	
Insyte^®^	16	22.2	359	31.0	375	28.4	
Intima^®^	3	4.2	75	6.5	78	5.9	
**Catheter gauge** (n = 1,263)							0.805^b^
16 gauge	0	0.0	1	0.1	1	0.1	
18 gauge	0	0.0	5	0.4	5	0.4	
20 gauge	4	5.1	91	7.7	95	7.5	
22 gauge	63	79.7	889	75.0	952	75.4	
24 gauge	12	15.2	198	16.7	210	16.6	
**Laterality of the insertion site** (n = 1,318)							0.365^a^
Right	42	52.5	7144	57.7	756	57.3	
Left	38	47.5	524	42.3	562	42.6	
**Insertion site** (n = 1,316)							0,054^a^
Forearm	40	50.0	633	51.2	673	51.1	
Back of hand	13	16.3	95	7.7	108	8.2	
Antecubital fossa	7	8.8	122	9.9	129	9.8	
Wrist	10	12.5	135	10.9	145	11.1	
Arm	10	12.5	251	20.3	261	19.8	
**PIC method of use** (n = 1,304)							0.065^a^
Intermittent	68	85.0	930	76.0	998	76.5	
Continuous	12	15.0	294	24.0	306	23.5	
**PIC method of removal**							0.184^a^
96H	45	56.2	602	48.6	647	49.1	
Clinical indication	35	43.8	637	51.4	672	50.9	
**Pain complaint**							<0.001^a^
No	26	32.5	984	79.4	1.010	76.6	
Yes	54	67.5	255	20.6	309	23.4	
**Use of antibiotic therapy**							0.102^a^
No	22	27.5	453	36.6	475	36.0	
Yes	58	72.5	786	63.4	844	64.0	

^a^ Chi-square independence test, ^b^Fisher’s exact test.

Despite the small number in some analyses, it was identified that the use of some drugs was significantly associated with the development of phlebitis, such as Bromopride (p = 0.012), Ketoprofen (p = 0.012), <0.001), Enoxaparin (p < 0.001), Furosemide (p < 0.001), Sodium Heparin (p < 0.001), Hydrocortisone Sodium Succinate (p = 0.012), Metronidazole (p < 0.001), Omeprazole Sodium (p = 0.013), Ondansetron Hydrochloride (p = 0.001), Tramadol Hydrochloride (p = 0.002).

The diagnostic curve of the classification model used was the ROC curve, and the area under the curve was 84.0%, the model’s success rate was 94.0%, the specificity was 99.7%, and the sensitivity 6.25%, with 94.0% of the variables well classified, with their study being used in the analyses detailed below.

According to Table 3, when modeling logistic regression of univariate analysis considering only the variables with p-value <0.20 in the chi-square test of Tables 1 and 2, the significance of reduced mobility, insertion of PIC in veins in the hand region, and pain complaints are highlighted as risk indicators for the occurrence of phlebitis. Moreover, regarding drugs, Amoxicillin-Potassium Clavulanate (p = 0.002) and Omeprazole Sodium (p = 0.002) stood out in the analysis model.

**Table 3.** Univariate analysis using the logistic regression model of patients with or without peripheral intravenous catheters-related phlebitis, according to the variables of interest selected in the association test. São Paulo, Brazil, 2020.

Variable	Estimate	Error	p	OR	CI 95% OR
PIC method of removal	–0.31	0.23	0.185	0.74	(0.47; 1.16)
Reduced mobility	0.6	0.2	0.012	1.8	(1.14; 2.89)
VTE risk^1^	0.5	0.3	0.074	1.7	(0.95; 3.09)
Family history of VTE^1^	2.1	1.2	0.094	7.8	(0.7; 87.27)
Insertion site – Back of the hand	0.8	0.3	0.022	2.2	(1.12; 4.2)
Pain	2.1	0.2	<0.001	8.0	(4.92; 13.05)
Use of ATB^2^	0.4	0.3	0.104	1.5	(0.92; 2.52)
Amoxicillin-Potassium Clavulanate	1.9	0.6	0.002	6.5	(1.98; 21.1)
Omeprazole Sodium	0.7	0.2	0.002	2.0	(1.28; 3.2)

^1^ Venous thromboembolism, ^2^Antibiotic.

Based on the multivariate analysis final model (Table 4), the selected variables were those presenting p < 0.20 in the univariate regression analysis in Table 3; the interpretation of the coefficients of the final model by the OR, in relation to patients who had reduced mobility, showed that the chance of having phlebitis was 1.8. Those with family history of VTE had 22.7. Furthermore, patients who had their PIC inserted in the back of the hand had a 2.8 times greater risk of developing phlebitis when compared to other insertion sites, and those who reported pain at PIC insertion had 8.3 times greater chance of developing phlebitis.

**Table 4.** Multivariate analysis – final model of factors associated with the occurrence of peripheral intravenous catheter-related phlebitis – São Paulo, Brazil, 2020.

Variable	Estimate	Error	p	OR	CI 95% OR
PIC method of removal	–0.31	0.23	0.185	0.74	(0.47; 1.16)
Reduced mobility	0.59	0.26	0.015	1.8	(1.08; 3.00)
Family history of VTE^1^	3.12	1.28	0.05	22.7	(1.84; 280.56)
Insertion site – Back of the hand	1.05	0.37	0.012	2.8	(1.39; 5.83)
Pain	2.12	0.26	<0.01	8.3	(4.98; 13.93)
Amoxicillin-Potassium Clavulanate	2.01	0.73	0.015	7.4	(1.8; 30.78)
Omeprazole Sodium	0.54	0.25	0.029	1.7	(1.05; 2.81)

^1^ Venous thromboembolism.

Among the drugs used by the patients studied, statistical significance was identified for the occurrence of phlebitis in those who used Amoxicillin-Potassium Clavulanate (5%; p = 0.015; OR 7.4) and Omeprazole Sodium (53.3%; p = 0.029; OR 1.7).

## DISCUSSION

The results identified showed that the proportion of patients who had phlebitis was 6.1%, among the 1,319 patients studied, exceeding the INS recommendation of a proportion of 5%^(1)^.

The literature on the subject indicates quite different frequencies of PIC-related phlebitis, ranging from 1.7% to 70%. This discrepancy arises, among others, as a result of the lack of standardization in the diagnosis, the monitoring, and the behavior, as well as of divergences in the condition classifications by professionals^(8,13)^. The present study, although having values slightly above the recommended, is inferior if compared to other national studies, which report incidences from 7.5%^(17)^ to 31.1%^(8)^.

Regarding the length of PIC permanence and the occurrence of phlebitis, in the primary study^(15)^ a significant difference was identified in the time from 24 to 48 hours, with the highest complication rate (7.3%), if compared to the other times evaluated. This finding is also evidenced in another study^(17)^ and relates the phlebitis event to a time of up to 48 hours due to the probable biological inflammatory response for patients prone to this complication.

Aiming at the pathophysiology (Virchow’s triad) related to VTE, the relationship between its risk factors and the development of phlebitis was investigated. It was identified that family history of VTE was a risk factor for phlebitis, as well as being an indicator for other clinical complications. These findings corroborate the study^(18)^, in which the specific biological susceptibility to develop phlebitis was correlated with the risk of VTE. Furthermore, among the risk factors for VTE, reduced mobility was also shown to have a significant correlation, either temporary or permanent mobility reduction, with the probability of developing phlebitis, as well as other complications^(19)^. Based on the study of the Maddox scale, used to identify and classify phlebitis severity, reduced mobility was one of the main nursing diagnoses (72.2%) identified^(18)^.

Regarding PIC insertion site, the back of the hand was the one with the highest risk for the development of phlebitis, in the univariate and multivariable analyses. Despite the frequent use of veins in this region for PIC insertion in clinical practice in Brazil, studies conducted in other countries and showing a high proportion of phlebitis when using PIC in this region, 23.8%^(8)^ and 36.5%^(20)^, culminated in changes in good practice guidelines, contraindicating PIC insertion in adults in veins of the back of the hand^(21)^. Regarding the catheter gauge, no effect was identified in relation to the occurrence of phlebitis, a finding that was also present in former studies.

It is noteworthy, therefore, that the latest scientific evidence supports a change in the classic nursing behavior in initiating peripheral venipuncture for PIC insertion, from the most distal to the proximal vessels, including in this proposal the veins of the dorsal arch of the hands. Thus, the inclusion of prioritization of the most distal vessels of the forearm for vascular access in nursing care protocols is supported, aiming to control the risk of development of PIC-related phlebitis in veins in the arch of the back of hand^(20)^.

It was observed that 67.5% of the patients complained of pain at the PIC insertion site, with no association with signs of inflammation. What is observed in care practice is that, in most cases, this complaint is not valued as an indication for PIC withdrawal, if not associated with other clinical indicators. It should be noted that our result showed that patients who reported pain on PIC insertion site were 8.3 times more likely to develop phlebitis. It is worth highlighting that, even in the INS Phlebitis Scale^(1,16)^, pain is not an absolute factor for the diagnosis of milder phlebitis, Grade I. Erythema is the one to determine the beginning of phlebitis on this scale, and the pain may or may not be present. Thus, the need to value pain as an early indication of the possibility of development of phlebitis, even without the presence of erythema, is highlighted, especially for nursing teams that use the INS Phlebitis Scale^(1,16)^ in clinical practice.

It is known that chemical phlebitis can be directly related to the administration of drugs with extremes of pH, osmolarity, and/or with substances that irritate the vessels. The administration of drugs with extreme pH is related to the occurrence of phlebitis, because the more acidic or basic the drug, the greater the risk of chemical phlebitis^(8,17)^. Moreover, some drugs used during IVT in some institutions are not recommended for administration through PIC in others, with the need to conduct in-depth studies for the prevention of chemical phlebitis^(15,22)^.

The analyses carried out showed that the occurrence of phlebitis was associated with the use of some drugs. To minimize this occurrence, specific care is needed in the preparation and, in particular, in increasing drug dilution and infusion time, as well as in the continuous evaluation of PIC insertion site during its infusion^(3,9,23)^. National studies found significant associations^(3,17)^, corroborating this finding, revealing that the increased risk of chemical phlebitis is associated with the administration of antimicrobial drugs, in view of the specific pharmaceutical properties and possible technical failures in their preparation and administration^(3,9,24)^.

In this setting, the need for nurses to approach clinical pharmacists, as well as the pharmaceutical industry, so that drug and parenteral solutions manufacturing technologies can be more synergistic with the needs of vascular health preservation, as well as with directing preventive measures for chemical phlebitis.

As for the analysis of the variable use of antibiotics, in the present study, there was a significant association of the occurrence of phlebitis with the use of Amoxicillin-Potassium Clavulanate. An association was observed with the study^(9)^ that demonstrated that the administration of Amoxicillin-Potassium Clavulanate resulted in greater potential for the development of phlebitis. It should be noted that the fact that Amoxicillin-Potassium Clavulanate presented a pH between 8.0 and 10 may have increased the risk for the occurrence of chemical phlebitis^(22,25)^. Other authors state that antibiotics with similar characteristics were considered a risk factor for the development of this event^(3,9,12,25)^.

Omeprazole Sodium has a pH between 8.8 and 9.2 and demonstrated that its use corroborated 1.7 times greater chance of phlebitis. The analysis of the extremely basic hydrogen potential profile of this drug can support the finding. Furthermore, it is also consistent with the findings of the study^(26)^ on incidence and associated factors, which found association with drugs of similar characteristics.

Additionally, it is noteworthy that no significant correlation was found between phlebitis and age and female sex. The results related to sex and age are similar to a more recent one^(9)^ that also did not obtain associations with phlebitis.

At the end of this study, we can emphasize that phlebitis is a multifactorial event and different strategies are required for its identification and prevention, from choosing the best device and gauge suitable for the patient and therapy prescribed, insertion site, as well as the early detection of phlebitis, possible through systematic clinical assessment and audit of compliance monitoring, as well as the use of technologies, such as ultrasound, and the dilution and administration of evidence-based drugs, with the objective of reducing the occurrence of this complication.

Additionally, it is noted that there are opportunities for improvement in the attention of the pharmaceutical industry in what regards the formulation and physicochemical behavior of drugs that are more synergistic with biological needs to maintain vascular health.

One of the limitations of this study is the performance of secondary analysis research, subject to the influences of selection biases and inclusion of the sample according to the objectives of the main study. However, it should be highlighted that the robust sample of this investigation supports the institution of measures to prevent PIC-related phlebitis in terms of promoting patient mobility, non-insertion of PIC in veins of the back of the hand, cautious infusion of drugs, and valuing the isolated complaint of PIC-related pain as an indication of phlebitis.

## CONCLUSION

The risk factors in the multivariable analysis of risk assessment for the occurrence of phlebitis found in the present study were the presence of reduced mobility, family history of VTE, insertion of PIC in veins of the back of the hand, complaint of pain on PIC insertion, as well as the use of the drugs Amoxicillin-Potassium Clavulanate and Omeprazole Sodium.

## ASSOCIATE EDITOR

Thelma Leite de Araújo
